# Influence of Fly Ash–Ground Granulated Blast Furnace Slag Blends and PVA Fibres on the Dimensional Stability, Durability and Microstructure of Foamed Concrete

**DOI:** 10.3390/ma19173781

**Published:** 2026-09-05

**Authors:** Tengfei Ma, Pau Chung Leng, Bin Sha

**Affiliations:** 1 Faculty of Built Environment and Surveying, Universiti Teknologi Malaysia, Skudai 81300, Johor, Malaysia; pcleng2@utm.my; 2 College of Civil Engineering and Architecture, Jiaxing Nanhu University, Jiaxing 314001, China; shabin@jxnhu.edu.cn

**Keywords:** foamed concrete, supplementary cementitious materials, PVA fibre, dimensional stability, durability

## Abstract

**Highlights:**

FA–GGBS blends and PVA fibres were evaluated for foamed concrete performance. SCM replacement reduced chloride penetration but increased drying shrinkage. PVA fibres reduced drying shrinkage and freeze–thaw-induced strength loss. Multi-criteria evaluation identified mixtures with balanced overall performance. C0.3 ranked among the top three mixtures under all six weighting schemes.

**Abstract:**

Foamed concrete is susceptible to drying shrinkage and environmental deterioration because of its porous structure. This study evaluated the effects of fly ash–ground granulated blast-furnace slag blends and polyvinyl alcohol fibres on the dimensional stability and durability of FC. Sixteen mixtures with SCM replacement levels of 0–35% and PVA fibre contents of 0–0.3 vol.% were tested for accelerated drying shrinkage, freeze–thaw resistance, sulfate wet–dry resistance, chloride penetration, and visible pore structure. A normalised multi-criteria evaluation was used to compare the mixtures across five performance indicators, with sensitivity analysis performed for six weighting schemes. Increasing SCM replacement generally increased the drying shrinkage but reduced the chloride penetration depth by 26.7%, 33.3%, and 56.7% at 15%, 25%, and 35% replacement, respectively. At 25% SCM replacement, PVA fibres reduced freeze–thaw-induced compressive strength loss by 62.12–98.48% relative to the corresponding fibre-free mixture. Optical microscopy identified a local stratified region in one of three C0.3 specimens, which was not considered a systematic feature. C0.3 achieved the highest overall score under equal weighting and remained among the top three mixtures under all six weighting schemes. Within the investigated mixture range, C0.3 showed consistently high overall performance, although longer-term durability assessment is required.

## 1. Introduction

Foamed concrete (FC) is a lightweight cementitious material characterised by low density, good thermal insulation, and fire resistance [1]. These properties have supported its use in building insulation, prefabricated wall panels, floor filling, and geotechnical engineering applications [2,3]. Its cellular structure reduces structural weight while maintaining thermal insulation performance. Growing demand for energy-efficient and sustainable construction has further increased the use of FC as a lightweight construction material [4].

The porous structure that contributes to its low density also increases its susceptibility to drying shrinkage and environmental deterioration. Moisture loss during curing and service may cause shrinkage, leading to microcracking and reduced dimensional stability [5]. The interconnected pore network facilitates the ingress of water and aggressive ions, increasing vulnerability to freeze–thaw damage, sulfate attack, and chloride exposure [6]. Degradation of the cementitious matrix can consequently reduce mechanical performance and service life [7]. Improving dimensional stability and durability therefore remains a key challenge for the broader application of FC in engineering practice.

Supplementary cementitious materials (SCMs), particularly fly ash (FA) and ground granulated blast-furnace slag (GGBS), are widely incorporated into cement-based materials to reduce cement consumption and associated carbon emissions while enhancing durability. Pozzolanic and latent hydraulic reactions promote the formation of additional calcium silicate hydrate (C–S–H) gel and reduce calcium hydroxide content in the hardened matrix [8]. The fine particles of SCMs also fill interparticle voids, contributing to a denser microstructure and lower permeability [9]. These changes have been associated with improved resistance to chloride ingress, sulfate attack, and freeze–thaw deterioration. Binary SCMs systems containing both FA and GGBS have attracted particular interest because their complementary hydration characteristics often result in better long-term durability than single-SCMs systems [10,11].

The effect of SCMs on dimensional stability is less clear. Their relatively low early-age reactivity can delay the development of a rigid cementitious skeleton during curing when high replacement levels are used [12]. Although continued hydration refines the pore structure at later ages, an increase in fine capillary pores may generate higher capillary stresses during drying and lead to greater shrinkage deformation [13]. As a result, previous studies have reported inconsistent relationships between SCMs content and drying shrinkage, particularly in FC, whose cellular pore structure differs markedly from that of conventional concrete [14,15]. Most studies have examined either durability or shrinkage, whereas investigations addressing both aspects simultaneously remain limited.

Fibre reinforcement has been widely used to mitigate the limitations associated with SCMs incorporation. Among the fibres used in cement-based materials, polyvinyl alcohol (PVA) fibres have received considerable attention because of their high tensile strength, hydrophilic nature, and strong bond with cementitious matrices [16,17]. These characteristics enable PVA fibres to bridge microcracks, transfer tensile stresses across crack surfaces, and restrict crack propagation [18]. Previous studies have shown that PVA fibres can reduce drying shrinkage cracking and improve crack resistance. Improved freeze–thaw and sulfate resistance has also been reported [19,20], which is generally attributed to the ability of the fibres to limit crack opening and delay the development of interconnected damage pathways within the matrix [21,22].

Despite extensive research on SCMs and PVA fibres individually, their combined effects remain less well understood [23,24]. Most studies have examined either SCMs or fibres in isolation, whereas the combined influence of these materials on the microstructure and durability development of FC has received comparatively limited attention [25]. Although the effects of SCMs and PVA fibres on individual durability properties of FC have been reported [26], studies that simultaneously evaluate dimensional stability, multiple durability indicators, and overall performance remain scarce. The interaction between SCM-induced pore refinement and fibre-mediated crack control has also not been systematically investigated, despite its potential relevance to moisture transport and damage development.

Most existing studies have assessed drying shrinkage and durability properties independently [27]. However, dimensional stability, freeze–thaw resistance, sulfate resistance, and chloride resistance are governed by different deterioration mechanisms; therefore, improvement in one property does not necessarily correspond to improved overall durability [28]. Evaluation based on a single performance indicator consequently provides limited guidance for mixture design.

Although optimisation of multiple performance attributes is increasingly important in engineering practice, integrated assessment methods that consider both shrinkage behaviour and multiple durability indicators remain limited for FC. Systematic approaches for identifying mixture designs that achieve balanced overall performance, rather than maximising a single property, are therefore still lacking.

This study investigated the combined effects of FA–GGBS supplementary cementitious materials (SCMs) and PVA fibres on the dimensional stability and durability of FC. Sixteen mixtures with different SCM replacement levels and PVA fibre dosages were evaluated through accelerated drying shrinkage, freeze–thaw cycling, sulfate wet–dry cycling, chloride penetration, and optical microscopy. A normalised multi-criteria performance evaluation using best-value normalisation was applied to integrate five performance indicators and assess the overall performance of the mixtures. The sensitivity of the integrated evaluation to different weighting schemes was examined. The study aimed to assess the combined effects of SCMs and PVA fibres across the individual performance indicators and identify a mixture with balanced overall performance within the investigated mixture range.

## 2. Materials and Methods

### 2.1. Cementitious Materials

Ordinary Portland cement (OPC, P·O 42.5) conforming to GB 175–2023 was used as the primary binder. The cement had a density of 3.13 g/cm^3^ and compressive strengths of 25.2 MPa and 52.3 MPa at 7 and 28 days, respectively. Grade II FA and S95 GGBS, conforming to GB/T 1596–2017 and GB/T 18046–2017, respectively, were used as SCMs. The densities of FA and GGBS were 2.10 and 2.90 g/cm^3^, respectively. FA had a bulk density of 1.10 g/cm^3^, while GGBS had a specific surface area of 412 m^2^/kg. The main oxide compositions of OPC, FA, and GGBS are presented in Table 1.

### 2.2. PVA Fibres

PVA fibres supplied by Shanghai Chenqi Chemical Technology Co., Ltd. were used as reinforcement. The fibres had a length of 12 mm, a nominal diameter of 15.5 μm, a density of 1.29 g/cm^3^, a tensile strength of 1830 MPa, and an elastic modulus of 40 GPa. To minimise fibre agglomeration, they were gradually added to the cementitious slurry during mixing.

### 2.3. Foaming Agent

A commercially available protein-based foaming agent (PFA) supplied by Shandong Meijiatu New Material Technology Co., Ltd. was used to produce preformed foam. The foaming agent was diluted with water at a volume ratio of 1:30 before foaming.

### 2.4. Mix Proportions

Sixteen FC mixtures with a target dry density of 1000 kg/m^3^ were prepared in accordance with the Chinese standard *Technical Specification for Application of Foamed Concrete* (JGJ/T 341–2014). All mixtures were produced with a constant water-to-binder ratio (*w*/*b*) of 0.40 and a constant total binder content. CF (calcium formate) was used as an accelerator, PCE (polycarboxylate ether superplasticizer) as a superplasticizer, and HPMC (hydroxypropyl methylcellulose) as a foam stabiliser.

A binary SCMs system comprising FA and GGBS at a mass ratio of 1:1 was used to replace OPC partially. Replacement levels of 0%, 15%, 25%, and 35% (by mass of total binder) were investigated. PVA fibres were incorporated at volume fractions of 0%, 0.1%, 0.2%, and 0.3%. Detailed mixture proportions are given in Table 2.

### 2.5. Experimental Procedure

FC was prepared using the preformed foam method. OPC, FA, GGBS, and PVA fibres were first dry-mixed in a planetary mixer to ensure uniform distribution of the solid constituents. A PCE was dissolved in part of the mixing water and added to the dry blend. The remaining water, containing HPMC and CF, was then introduced, and mixing continued until a homogeneous cementitious slurry was obtained.

The protein-based foaming agent was diluted with water at a volume ratio of 1:30 and processed using a foam generator to produce stable preformed foam. The preformed foam had a density of 60 kg/m^3^. The generated foam was immediately transferred to the mixer and incorporated into the cementitious slurry to minimise the time between foam generation and mixing. The same foam-generation and mixing procedures were used for all mixtures. After foam incorporation, mixing continued under the specified conditions until a visually uniform mixture was obtained.

**Figure 1:**
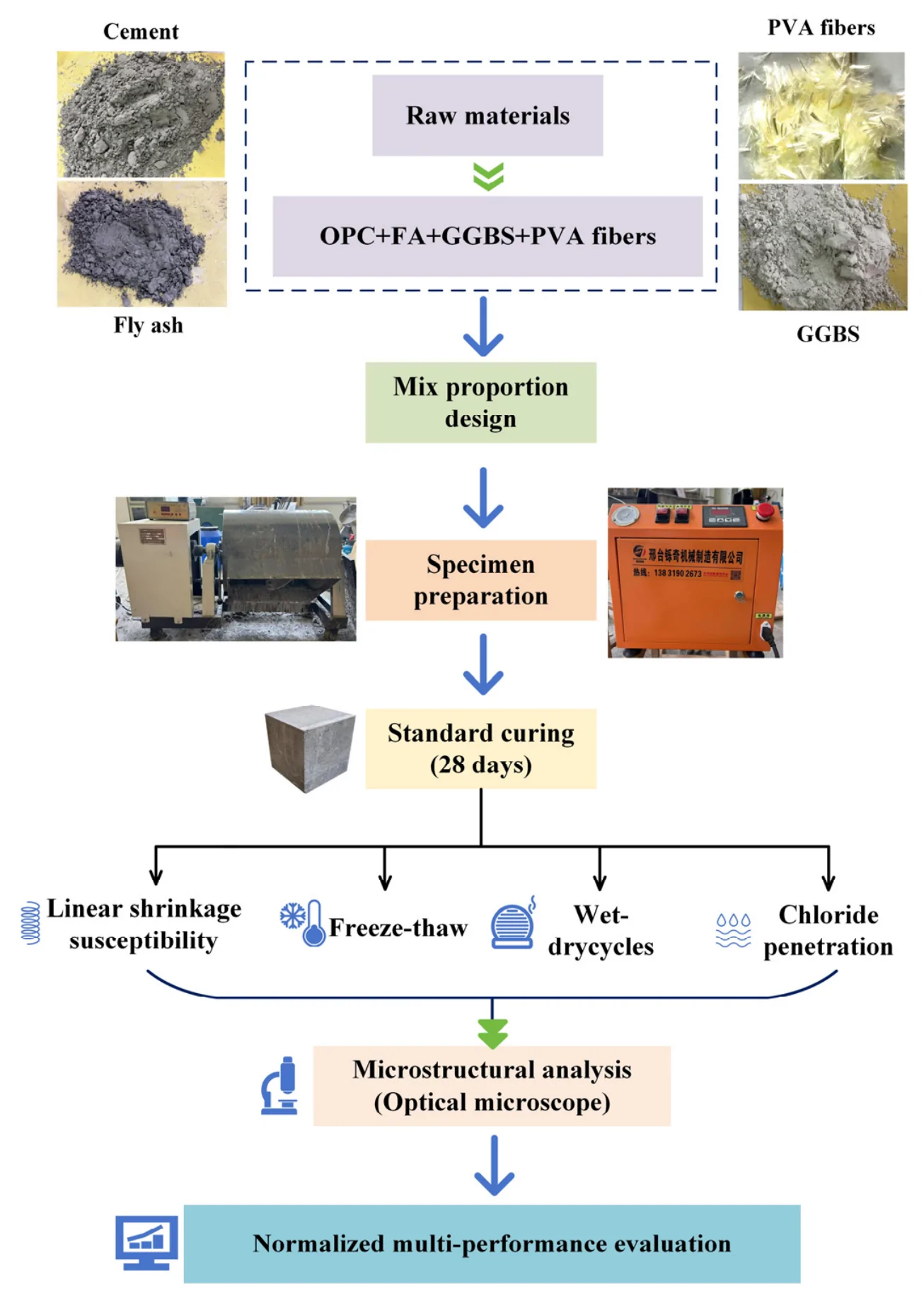
Experimental design and evaluation framework for FC.

The fresh FC was cast into moulds of the required dimensions for each test. After casting, the specimens were sealed for 24 h, demoulded, and cured under standard conditions (20 ± 2 °C and relative humidity ≥ 95%) for 28 days before testing. The overall experimental procedure is shown in Figure 1.

### 2.6. Assessment of Linear Shrinkage Sensitivity Under Accelerated Drying Conditions

Linear shrinkage under accelerated drying conditions was evaluated using an accelerated drying procedure. Prismatic specimens (40 mm × 40 mm × 160 mm) were demoulded after 24 h and cured under standard conditions (20 ± 2 °C and relative humidity ≥ 95%) for 28 days. Before drying, the initial length of each specimen was measured using a BY-160 length comparator. The specimens were then dried in a ventilated oven at 60 ± 2 °C for 24 h, after which the final length was measured using the same instrument. The accelerated drying condition was adopted to facilitate a comparative evaluation of the shrinkage response of the different mixtures within a practical testing period.

Linear shrinkage was calculated from the ratio of the measured length change to the nominal specimen length (160 mm), as given in Equation (1).

(1) $$S=\frac{∆L}{160}\times 1000$$

where S is the linear shrinkage rate (mm/m) and ΔL is the change in specimen length before and after accelerated drying (mm). Three specimens were tested for each mixture, and the mean value was reported.

### 2.7. Durability Evaluation

Unless otherwise stated, all durability tests were conducted after 28 days of standard curing. Three specimens were tested for each mixture, and the mean values were reported.

#### 2.7.1. Freeze-Thaw Test

The freeze–thaw resistance of foamed concrete was evaluated in accordance with the Chinese standard JGJ/T 341–2014. Clause 3.1.4 of the standard specifies a freeze–thaw resistance grade of D15 for the foamed concrete considered in this study. Accordingly, 15 freeze–thaw cycles were applied to the specimens. Each freeze–thaw cycle consisted of the following procedures: the specimens were first dried in an oven at 60 ± 5 °C to constant mass, cooled to room temperature, and then immersed in water at 20 ± 5 °C for 48 h. The specimens were then placed in a freezer at −20 ± 2 °C for 6 h, followed by thawing in water at 20 ± 5 °C for 5 h. This procedure was repeated until 15 cycles were completed. After 15 cycles, the mass loss and compressive strength loss of the specimens were determined. All mixtures were tested under the same conditions to enable comparison of their relative freeze–thaw resistance.

The reduction in freeze–thaw-induced compressive strength loss associated with PVA fibre incorporation was calculated relative to the corresponding fibre-free mixture at the same SCM replacement level, as shown in Equation (2).

(2) $$C_{i}=\frac{R_{i,0}-R_{i,PVA}}{R_{i,0}}$$

where *R*_i,0_ is the compressive strength loss of the mixture without PVA fibres at a given SCM replacement level, and *R*_i,PVA_ is the corresponding value for the PVA fibre-reinforced mixture.

#### 2.7.2. Sulfate Wet–Dry Test

The sulfate resistance of foamed concrete was evaluated using the wet–dry cycling method, in accordance with the Chinese *Standard for Test Methods of Long-Term Performance and Durability of Ordinary Concrete* (GB/T 50082–2024). Table 14.0.2 of the standard specifies exposure levels ranging from 15 to 150 wet–dry cycles. The 15-cycle exposure level (KS15) was selected for this study. The specimens underwent repeated wet–dry cycles under the specified sulfate exposure conditions. Each cycle consisted of immersion in a 5% sodium sulfate solution for 15 h, air drying for 1 h, and oven drying at 80 °C for 6 h. After 15 cycles, the compressive strength loss of the specimens was determined. The same test parameters were applied to all mixtures.

The 15-cycle exposure level was adopted as the minimum level specified in GB/T 50082–2024. This level was selected to compare the relative performance of the mixtures under controlled laboratory conditions rather than to reproduce the duration or severity of long-term field sulfate exposure.

#### 2.7.3. Chloride Penetration Test

**Figure 2:**
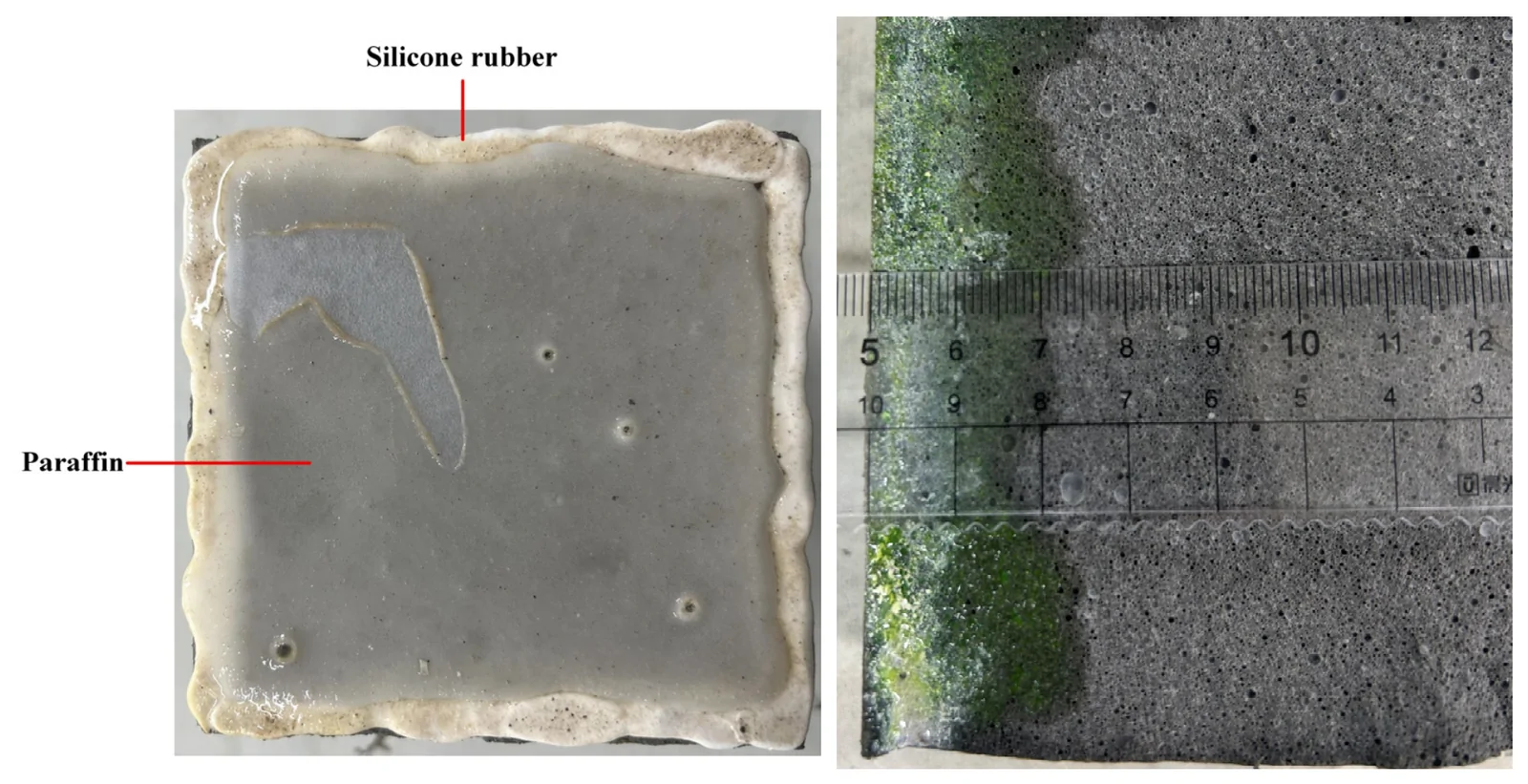
Chloride ion permeation experiment on FC.

Resistance to chloride ingress was evaluated in accordance with the Chinese standard *Test Code for Polymer-Modified Cement Mortar* (DL/T 5126–2021). Specimens were immersed in a 2.5% sodium chloride (NaCl) solution for 28 days and then split axially. A 0.1 mol/L silver nitrate solution and a 0.1% fluorescent indicator were applied to identify the chloride penetration zone. Penetration depth was determined from measurements taken at three equally spaced locations along each of the two boundaries between the fluorescent and uncoloured zones to the corresponding exposed surface. The average of the six measurements was reported as the chloride penetration depth. The specimen sealing procedure and test configuration are shown in Figure 2.

### 2.8. Normalised Multi-Criteria Performance Evaluation

To compare the overall performance of the FC mixtures, a normalised multi-criteria evaluation was conducted using five indicators: accelerated drying shrinkage, freeze–thaw resistance evaluated by mass loss and compressive strength loss, sulfate wet–dry cycle resistance, and chloride penetration resistance. Because these indicators have different units and numerical ranges, optimal-value normalisation was applied to each indicator before aggregation.

For indicators in which lower values represent better performance, the normalised score was calculated using Equation (3).

(3) $$I_{i}=\frac{X_{min}}{X_{i}}$$

For indicators in which higher values represent better performance, the normalised score was calculated using Equation (4).

(4) $$I_{i}=\frac{X_{i}}{X_{max}}$$

where *I_i_* is the normalised score of the i-th mixture, *X_i_* is the measured value of the corresponding performance indicator, and *X*_min_ and *X*_max_ represent the minimum and maximum values obtained in all mixtures, respectively. The normalised scores range from 0 to 1, with higher values indicating better performance.

Under the baseline equal-weighting scheme, each of the five indicators is assigned a weight of 0.20. The overall performance score is calculated as shown in Equation (5).

(5) $$S_{i}=\sum_{i=1}^{5}w_{i}I_{i}$$

where *S*_i_ is the overall performance score of mixture *i*, *w_i_* is the weight assigned to indicator *i*, and ∑*w_i_* = 1. Under equal weighting, this equation is equivalent to the arithmetic mean of the five normalised scores.

To examine the sensitivity of the overall evaluation to the weighting scheme, five additional scenarios were considered. In each scenario, one indicator was assigned a weight of 0.40 and the remaining four were assigned weights of 0.15. This resulted in six weighting schemes, including the baseline equal-weighting scheme. Radar charts were used to illustrate the normalised performance profiles of selected representative mixtures, while the overall performance scores were used to compare the integrated performance of all 16 mixtures.

## 3. Results

### 3.1. Dimensional Stability Under Accelerated Drying Conditions

**Figure 3:**
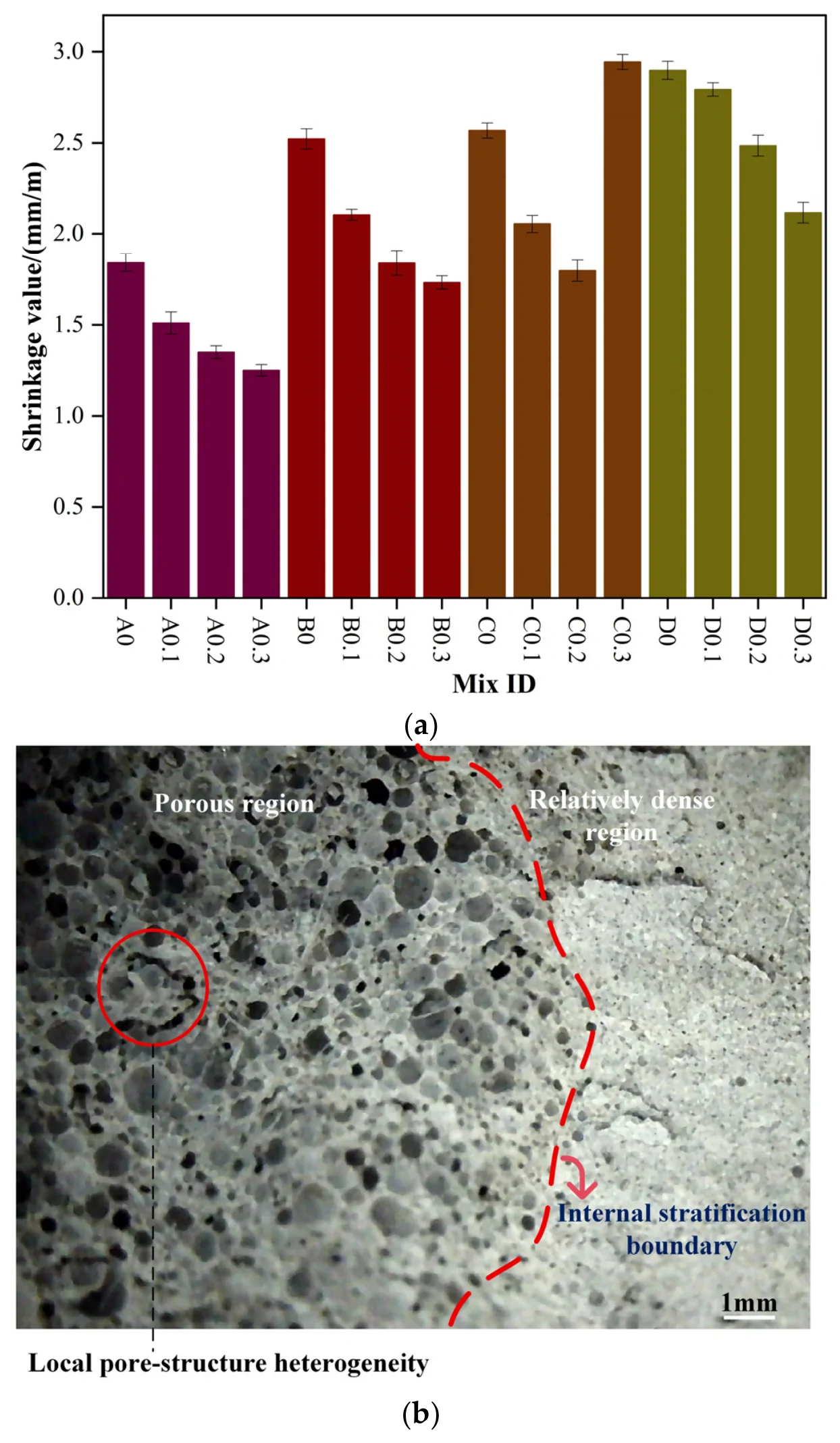
(**a**) Length change of foamed concrete specimens under accelerated drying conditions; (**b**) Optical micrograph of one C0.3 specimen showing local pore-structure heterogeneity.

Figure 3a shows that, in the absence of PVA fibres, linear shrinkage increased with an increasing SCMs replacement level. This trend may be associated with the lower early-age reactivity of FA and GGBS compared with ordinary Portland cement, which can delay the development of a rigid cementitious skeleton and increase deformation during accelerated drying [29,30]. SCMs incorporation also refines the pore structure by increasing the proportion of fine capillary pores [31]. Although total porosity may be reduced, a finer pore system can generate higher capillary stresses during drying, contributing to greater shrinkage [32]. Similar observations have been reported for cementitious materials containing FA and slag [33,34].

PVA fibre incorporation reduced linear shrinkage at all SCMs replacement levels [35]. The randomly distributed fibres formed a three-dimensional reinforcing network that restrained matrix deformation during drying [31]. The strong bond between PVA fibres and the surrounding hydration products also facilitated stress transfer, while fibre bridging limited the initiation and propagation of shrinkage-induced microcracks [36]. Consequently, mixtures containing higher PVA fibre contents generally exhibited improved dimensional stability.

The relatively high drying shrinkage of C0.3 was further examined using optical microscopy. Three C0.3 specimens were examined. One exhibited a distinct internal stratified region, characterised by a transition from a highly porous region to a relatively dense region (Figure 3). The visually identified boundary is marked by the dashed line in Figure 3. The other two specimens showed no comparable stratification. The stratification was therefore considered a local structural feature rather than a systematic characteristic of the C0.3 mixture or a direct consequence of the 0.3% PVA fibre dosage.

The local pore-structure heterogeneity may have contributed to the relatively high shrinkage of C0.3 by causing non-uniform moisture loss and deformation within the specimen. However, because the stratification occurred in only one of the three specimens, the optical microscopy results do not establish a direct causal relationship between the stratification and the measured shrinkage. The relatively high shrinkage of C0.3 should therefore be interpreted in the context of specimen-to-specimen variability rather than attributed solely to the stratified region. The reported shrinkage value was calculated from three independently tested specimens, with the corresponding standard deviation representing the measured variability.

The accelerated drying procedure was used to compare shrinkage responses rather than to predict long-term drying shrinkage under ambient conditions. Unlike standard methods such as ASTM C157, this procedure exposed the specimens to 60 ± 2 °C for 24 h, thereby accelerating moisture loss during the test period. A relationship between the measured accelerated shrinkage and long-term ambient drying shrinkage was not established because corresponding long-term measurements were not conducted. The effects of the elevated temperature on hydration and microstructural development also cannot be completely excluded. The shrinkage results are therefore interpreted as comparative responses under the adopted accelerated drying condition.

### 3.2. Freeze–Thaw Resistance

**Figure 4:**
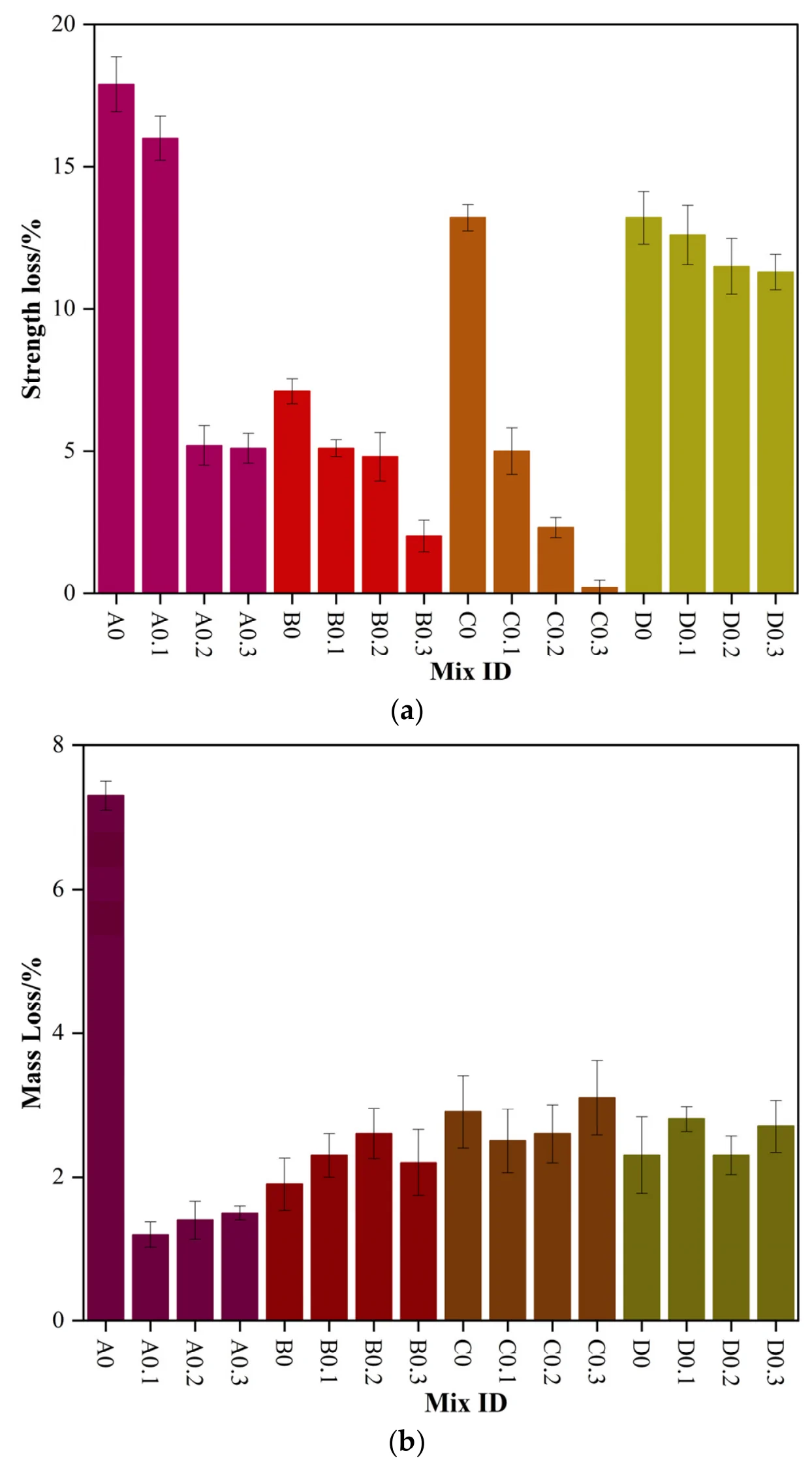
(**a**) The loss of compressive strength of FC after 15 freeze-thaw cycles; (**b**) The loss of mass of FC after 15 freeze-thaw cycles.

Figure 4a presents the compressive strength loss of FC after 15 freeze–thaw cycles. Relative to the control mixture (A0), mixtures containing SCMs generally exhibited lower strength loss, although no clear monotonic relationship was observed with increasing SCM replacement level. In most mixtures, increasing PVA fibre content further reduced strength loss, particularly at 15% and 25% SCM replacement. For example, the 25% SCM mixture showed lower compressive strength loss than A0 but higher loss than B0. This variation may be related to differences in pore characteristics and matrix development among the mixtures. Differences in cement-matrix development and visible pore structure may partly explain the variation in freeze–thaw response [37,38]. However, the present results do not establish a direct causal relationship between specific pore parameters and freeze–thaw degradation.

Figure 4b shows that A0 exhibited the highest mass loss after 15 freeze–thaw cycles, whereas all modified mixtures exhibited substantially lower mass loss, indicating less surface scaling. Differences among the modified mixtures were relatively small, with no consistent relationship with SCM replacement level or PVA fibre dosage. The mass-loss results therefore do not allow the individual contributions of SCMs and PVA fibres to surface deterioration to be distinguished. By comparison, the reduction in compressive strength loss showed a clearer association with PVA fibre incorporation, particularly at 25% SCM replacement. The improved retention of compressive strength may be related to the crack-bridging behaviour of PVA fibres, which can limit crack opening and damage propagation [39,40]. This interpretation is based on the measured macroscopic responses and reported crack-bridging behaviour of PVA fibres rather than direct observation of damage evolution during freeze–thaw cycling.

Overall, the two freeze–thaw indicators showed different patterns: mass loss differed little among the modified mixtures, whereas compressive strength loss showed a clearer association with PVA fibre incorporation. Taken together, the results do not support a consistent dependence of freeze–thaw performance on either the SCM replacement level or PVA fibre content alone.

The 15-cycle test used in this study corresponds to the D15 freeze–thaw resistance grade specified for foamed concrete in non-heated areas in JGJ/T 341–2014. The differences in the measured properties allowed the relative responses of the mixtures to be distinguished under the adopted 15-cycle conditions. These results should not, however, be interpreted as a direct assessment of long-term freeze–thaw durability or equated with the longer exposure periods specified in standards such as ASTM C666. Longer freeze–thaw exposure is required to establish the relationship between these results and long-term performance.

### 3.3. Chloride Penetration Resistance

**Figure 5:**
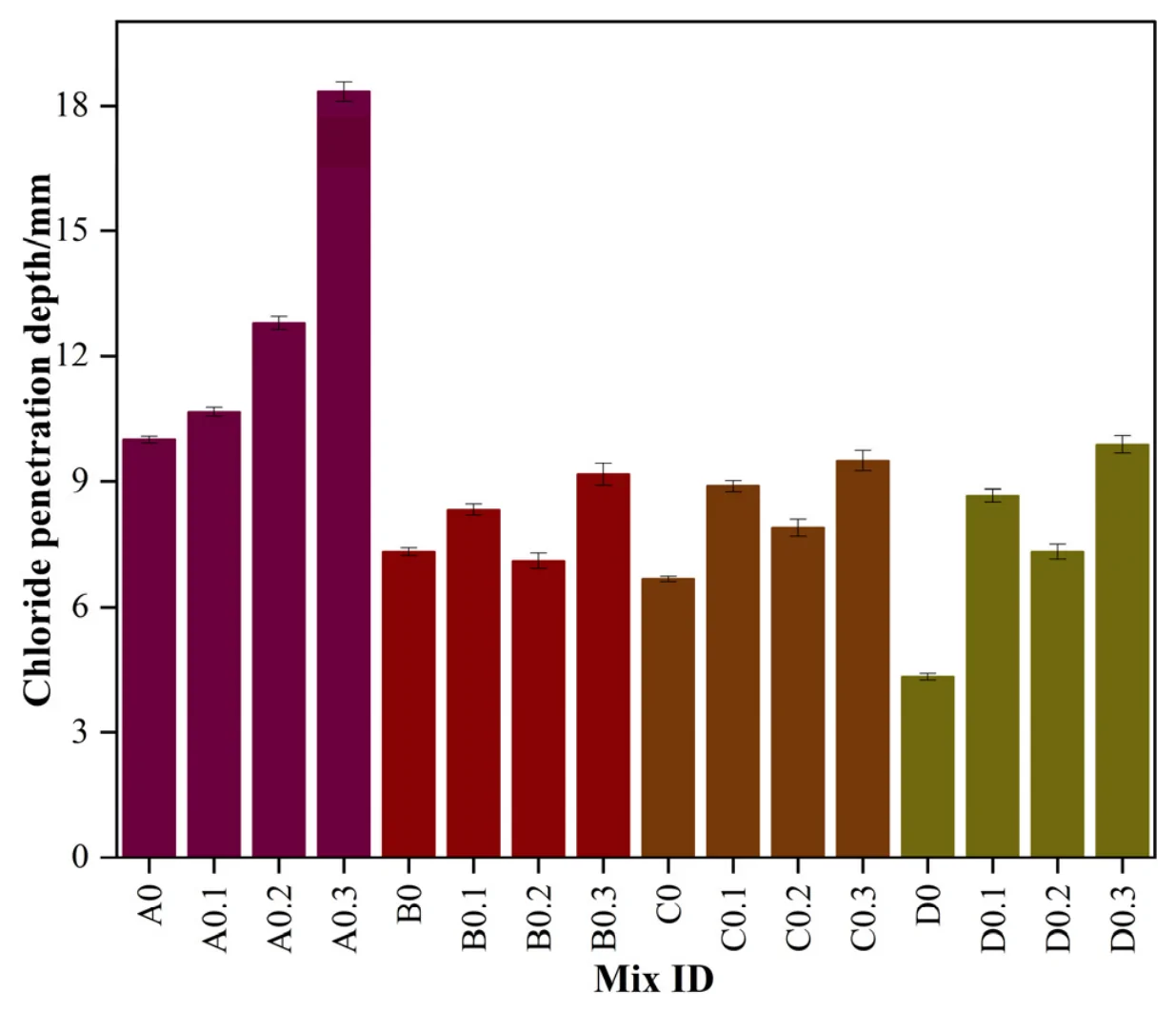
Chloride penetration resistance of FC.

Figure 5 presents the chloride penetration depth of FC after 28 days of immersion in a 2.5 wt.% NaCl solution. In mixtures without PVA fibres, chloride penetration depth decreased with increasing SCMs replacement level. Relative to the control mixture, penetration depth decreased by 26.7%, 33.3%, and 56.7% at 15%, 25%, and 35% SCM replacement, respectively. The corresponding mean values and standard deviations are shown in Figure 5. These results indicate improved resistance to chloride ingress with increasing SCM replacement.

The enhanced chloride resistance may be associated with the combined effects of FA and GGBS. The pozzolanic reaction of FA and the latent hydraulic reaction of GGBS can generate additional C–S–H gel, leading to pore structure refinement, reduced capillary pore connectivity, and increased tortuosity of chloride transport pathways. Such microstructural changes may hinder chloride ingress [41,42].

At a given SCMs replacement level, mixtures containing PVA fibres generally exhibited greater chloride penetration depths than the corresponding fibre-free mixtures. However, no clear monotonic relationship was observed between chloride penetration depth and PVA fibre dosage, indicating that the effect of PVA fibres on chloride transport cannot be explained by dosage alone. The hydrophilic nature of PVA fibres and fibre–matrix interfacial regions may alter local moisture and chloride transport pathways [43,44,45]. Fibre dispersion and fibre–matrix bond quality may further contribute to differences in local transport pathways among mixtures with different fibre contents [46]. These mechanisms were not directly characterised in the present study and therefore remain possible explanations rather than verified mechanisms.

Although PVA-fibre mixtures generally exhibited greater penetration depths, SCM-containing mixtures remained less penetrated than the corresponding control mixtures. Thus, the reduction associated with SCM incorporation was evident across the tested mixtures, whereas the response to PVA fibre incorporation varied with fibre dosage. This contrast indicates that SCM replacement and PVA fibre incorporation affected chloride penetration differently under the present test conditions. The potential influence of PVA fibre surface treatment on chloride transport warrants further investigation, particularly in relation to fibre–matrix interactions and local transport pathways.

The chloride penetration results also differed from the freeze–thaw response, for which PVA fibres reduced compressive strength loss after cyclic freezing and thawing. This difference may reflect the different transport and deterioration processes represented by the two tests. Freeze–thaw performance is influenced by damage development during repeated freezing and thawing [47], whereas chloride penetration reflects the transport of chloride-containing solution through accessible pore and transport pathways [48]. The present results therefore indicate that the improved freeze–thaw-related strength retention associated with PVA fibres does not necessarily correspond to lower chloride penetration.

### 3.4. Sulfate Wet–Dry Resistance

**Figure 6:**
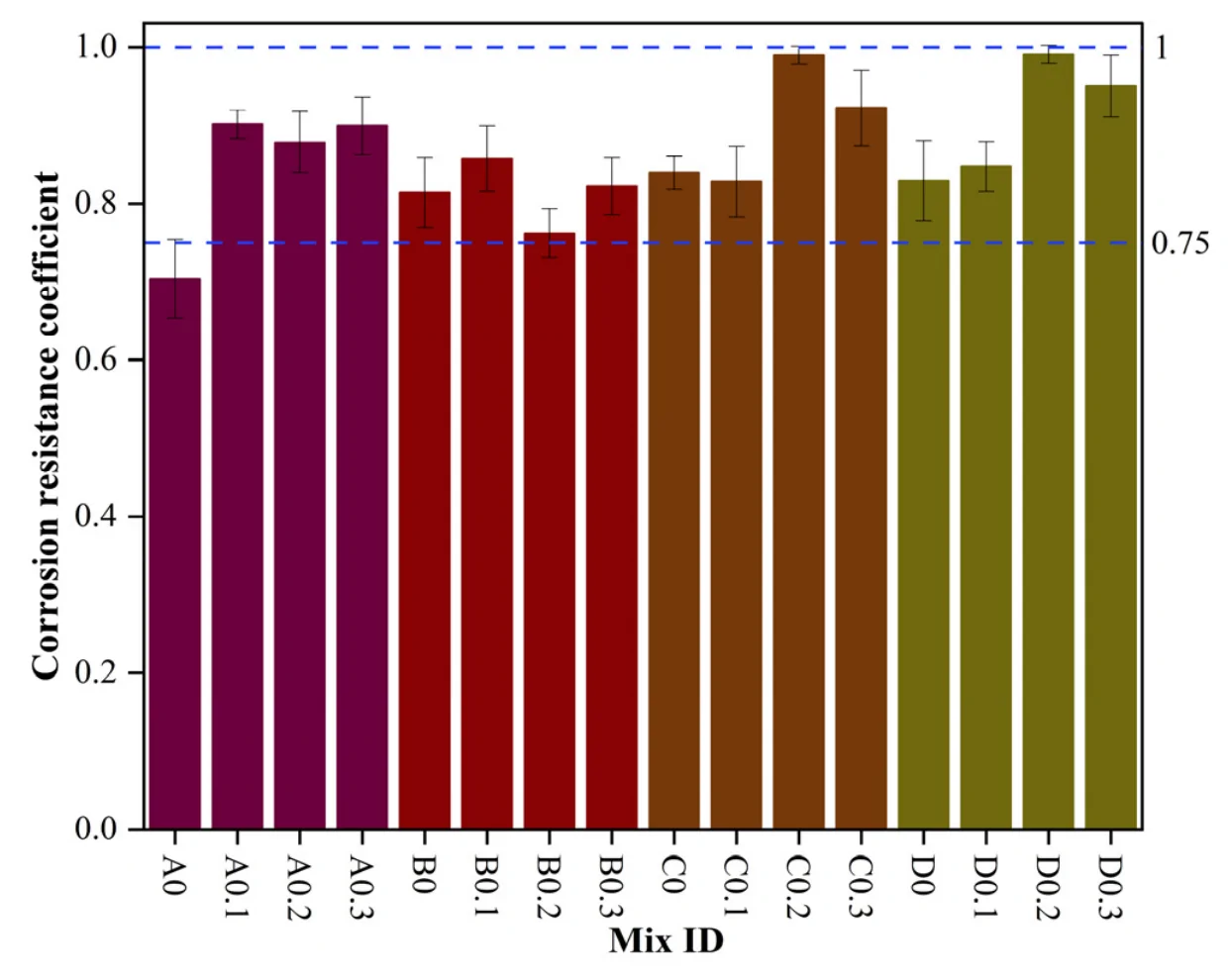
The tolerance of FC to 15 sulfate wet–dry cycles.

Figure 6 presents the corrosion resistance coefficient of FC after 15 sulfate wet–dry cycles. Relative to the control mixture, SCM-containing mixtures generally exhibited a higher corrosion resistance coefficient, while PVA fibre incorporation further increased the coefficient in most mixtures. However, neither SCM replacement level nor PVA fibre dosage showed a clear monotonic relationship with the corrosion resistance coefficient. The absence of a monotonic trend indicates that the response to sulfate wet–dry exposure varied among mixtures.

For example, the 35% SCM mixture had a higher corrosion resistance coefficient than the control mixture but a lower coefficient than the 25% SCM mixture. These differences may be related to changes in cement-matrix development and pore structure associated with SCM incorporation. A denser and more refined matrix may reduce pathways for sulfate solution ingress [49]. However, the present measurements do not directly establish the transport or reaction mechanisms underlying these differences.

T PVA fibre incorporation also affected the response to sulfate wet–dry exposure. For example, all three PVA-fibre mixtures in group D had higher corrosion resistance coefficients than D0, although the coefficient did not increase consistently with fibre dosage. The absence of a monotonic trend suggests that the response to PVA fibre incorporation may also depend on the interaction between fibre content and the surrounding cement matrix [50]. The effect of PVA fibres should therefore be interpreted from the measured degradation response rather than attributed solely to fibre content.

Overall, the combined use of SCMs and PVA fibres resulted in different corrosion resistance coefficients among the mixtures. The results indicate that SCM replacement and PVA fibre incorporation were associated with different responses to sulfate wet–dry exposure, although the present experiments cannot quantitatively distinguish the individual contributions of matrix modification and fibre reinforcement.

The 15-cycle exposure corresponds to the minimum sulfate wet–dry cycling resistance level (KS15) specified in Table 14.0.2 of GB/T 50082–2024. The selected exposure level allowed measurable differences among the mixtures to be identified and their relative responses to sulfate attack to be compared. However, the 15-cycle results should not be interpreted as a direct indicator of long-term sulfate resistance. The present study did not establish a relationship between the selected laboratory exposure conditions and long-term performance. Longer exposure periods are required to assess long-term sulfate resistance.

### 3.5. Optical Microscopic Observations

**Figure 7:**
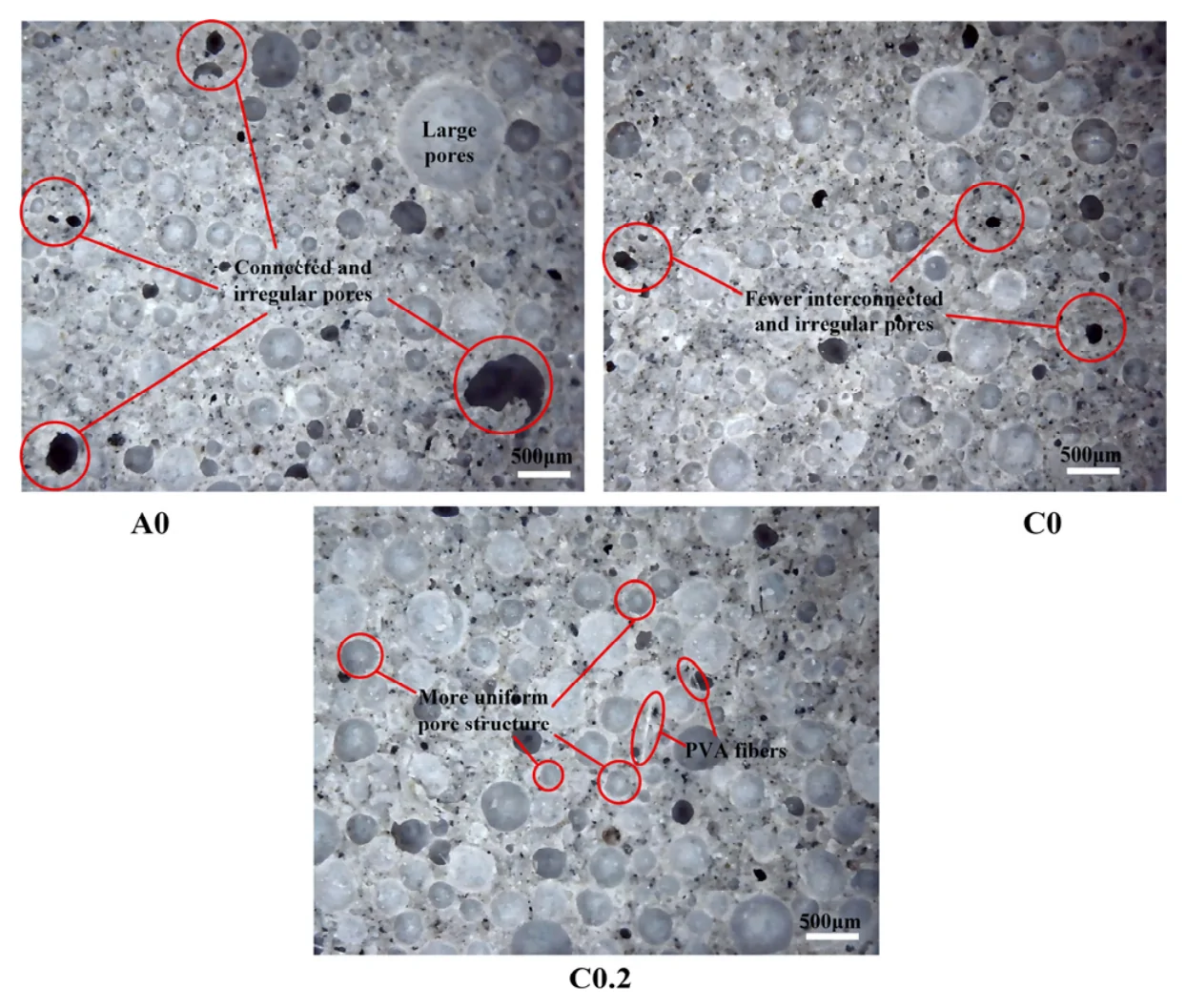
Optical microscopic observations of representative FC cross-sections.

To examine the relationship between pore structure and durability performance, representative optical micrographs of selected FC cross-sections are presented in Figure 7. Based on the normalised multi-criteria evaluation, three representative mixtures were selected for comparison: A0 (control), C0 (25% SCMs without PVA fibres), and C0.2 (25% SCMs and 0.2% PVA fibres).

The control mixture (A0) exhibited relatively large, irregular pores with pronounced interconnectivity. Local pore coalescence and non-uniform pore distribution were also observed, indicating limited pore stability during foam formation. Such features may facilitate moisture transport and the ingress of aggressive ions [51], consistent with the higher shrinkage, greater chloride penetration depth, and lower freeze–thaw and sulfate resistance reported in the preceding sections.

Compared with A0, specimen C0 exhibited a more uniform pore structure, characterised by smaller pores and fewer interconnected voids. The pore walls were also more continuous, suggesting improved pore uniformity following SCMs incorporation. These observations are consistent with the lower chloride penetration depth and improved durability performance reported in Section 3.1, Section 3.2, Section 3.3 and Section 3.4.

Among the mixtures investigated, specimen C0.2 exhibited the most uniform pore structure, with few oversized pores and a relatively even distribution of closed pores throughout the matrix. No obvious pore collapse or segregation was observed, indicating that the selected PVA fibre dosage did not adversely affect the stability of the foamed structure. This homogeneous pore structure was consistent with its superior overall durability performance [52].

The optical micrographs were consistent with the durability results. Mixtures with more homogeneous and less interconnected pore structures generally showed lower shrinkage, greater resistance to freeze–thaw and sulfate wet–dry cycling, and lower chloride penetration depths. These observations suggest that pore structure refinement was associated with the improved durability of the modified FC [53]. The incorporation of SCMs promoted a more uniform pore system, while an appropriate PVA fibre dosage helped maintain pore stability during foaming [54]. These modifications were associated with improved dimensional stability and overall durability.

### 3.6. Overall Durability Performance Evaluation Based on Normalised Multi-Criteria Analysis

To compare the mixtures across multiple performance indicators, the experimental results were normalised using the best-value method, with higher scores indicating better performance. The normalised data were used to construct radar charts for representative mixtures and to calculate overall performance scores for all 16 mixtures.

**Figure 8:**
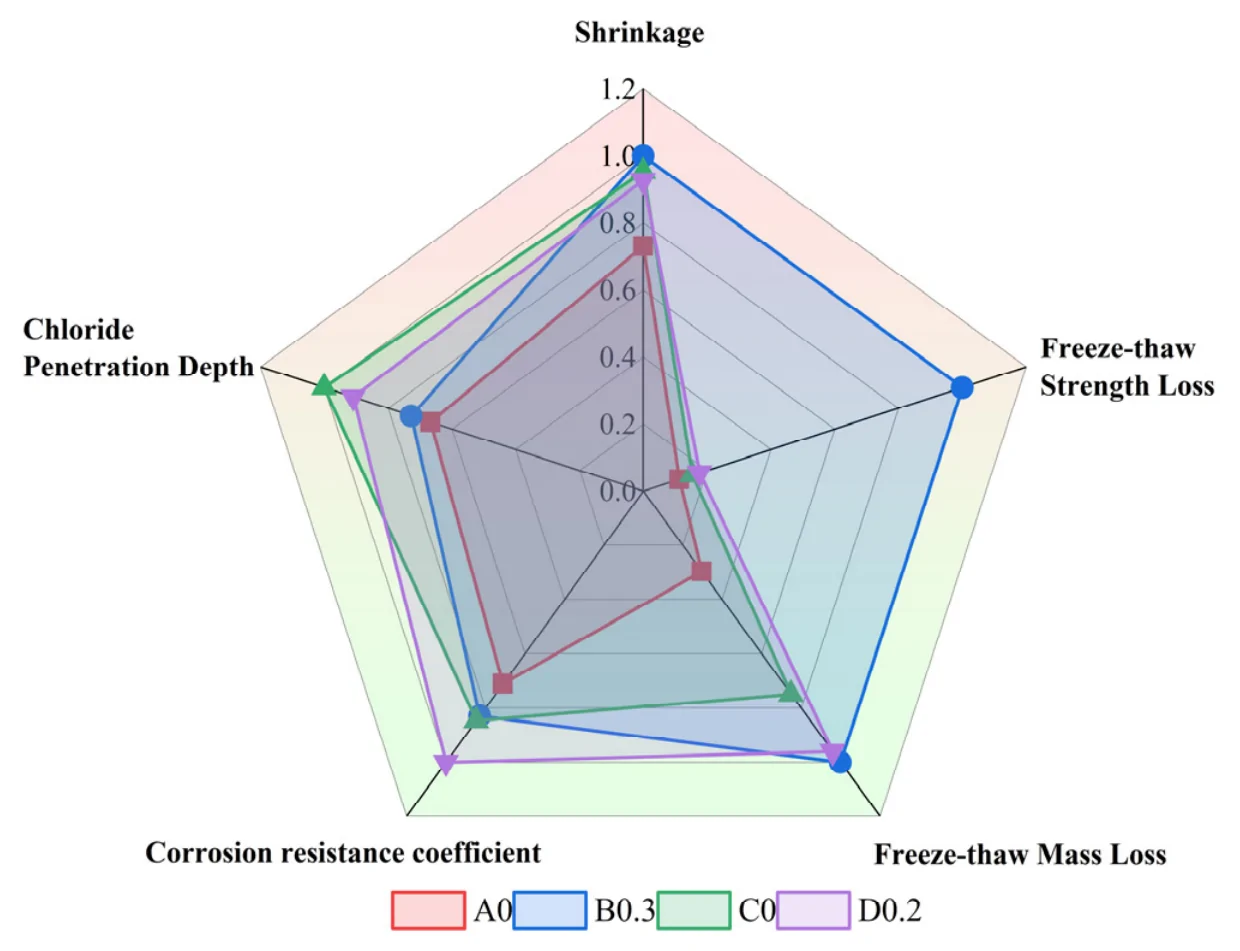
Normalised performance profiles of representative FC mixtures across the five indicators.

Figure 8 presents the normalised performance profiles of the representative mixtures across the five indicators. The radar charts illustrate the relative performance of the mixtures for individual indicators rather than provide an integrated ranking based on their visual shape or area. The control mixture (A0) generally exhibited lower normalised scores, whereas the modified mixtures showed improvements in different performance indicators. For example, D0.2 achieved a relatively high normalised score for sulfate resistance but lower scores for dimensional stability and freeze–thaw resistance. These differences illustrate trade-offs among the indicators and show that a high score for one indicator does not necessarily result in the highest overall performance.

**Figure 9:**
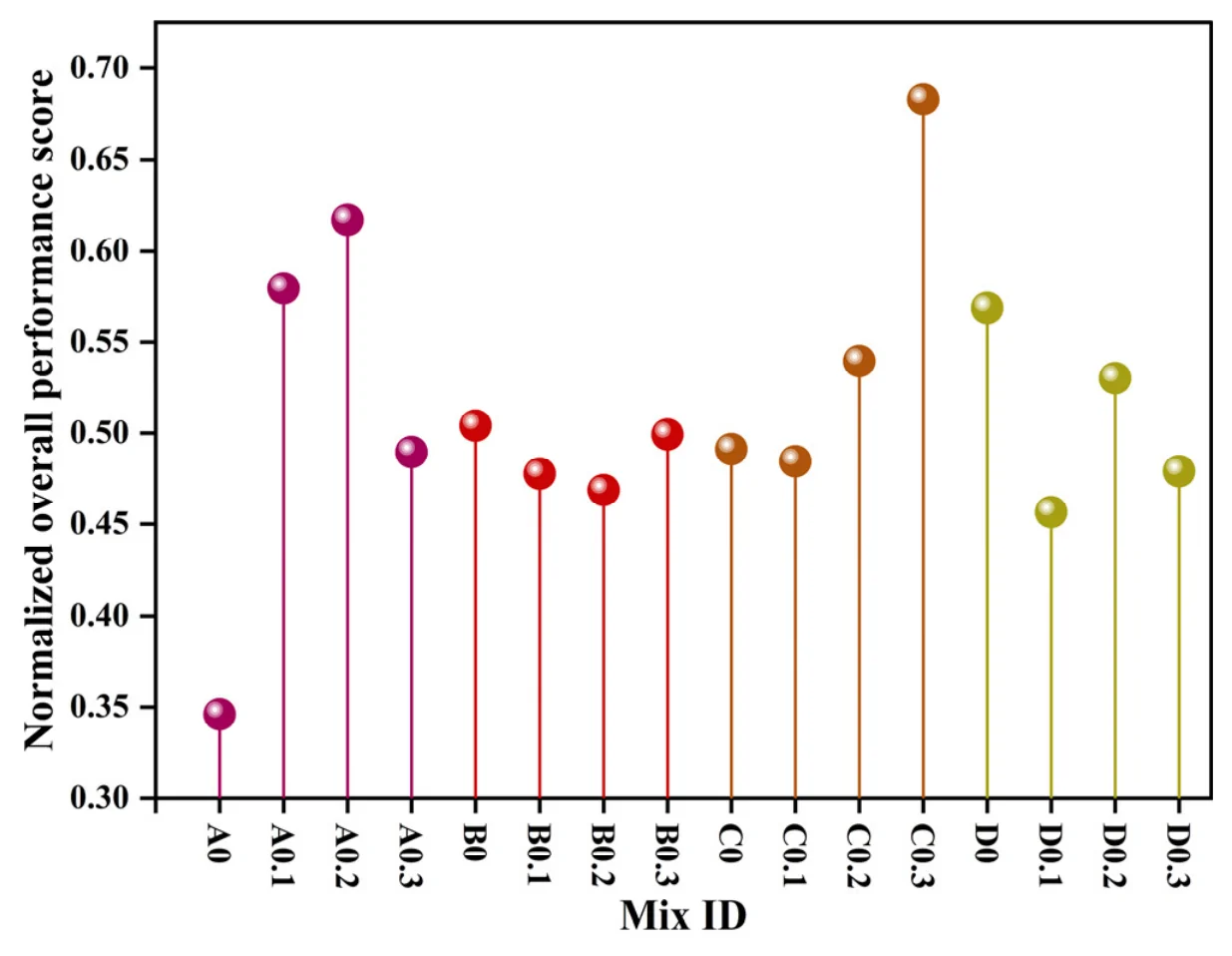
Overall performance scores of the 16 FC mixtures under equal weighting.

Figure 9 presents the overall performance scores of all 16 mixtures under the equal-weighting scheme, with a weight of 0.20 assigned to each indicator. C0.3 achieved the highest overall score under the equal-weighting scheme. The overall score did not vary monotonically with either SCM replacement level or PVA fibre dosage. The overall ranking therefore reflected the combined performance across the five indicators. A mixture with the highest score for an individual indicator therefore did not necessarily achieve the highest overall score.

Sensitivity to the weighting scheme was examined using six scenarios. The baseline scenario assigned a weight of 0.20 to each indicator. In each of the other five scenarios, one indicator was assigned a weight of 0.40 and the remaining four were assigned weights of 0.15. C0.3 ranked first under the equal-weighting scheme and also when freeze–thaw compressive strength loss or sulfate resistance was assigned a weight of 0.40. When drying shrinkage was assigned a weight of 0.40, A0.2 ranked first and C0.3 s. When freeze–thaw mass loss was assigned a weight of 0.40, A0.1 ranked first and C0.3 third. When chloride penetration depth was assigned a weight of 0.40, D0 ranked first and C0.3 s. The six weighting scenarios are summarised in Table 3. C0.3 remained among the top three mixtures in all six scenarios, although its ranking varied with the weighting of individual indicators.

Within the investigated mixture range, C0.3 (25% SCM replacement and 0.3 vol.% PVA fibres) had the strongest overall performance under the multi-criteria evaluation. It achieved the highest overall score under equal weighting and remained among the top three mixtures across all six scenarios.

Overall, the multi-criteria evaluation showed that the relative performance of the mixtures varied across the five indicators and depended on the weighting scheme. Mixture selection should therefore consider the relative importance of the individual indicators rather than rely on a single durability index.

## 4. Conclusions

The combined effects of FA–GGBS SCMs and PVA fibres on the dimensional stability and durability of foamed concrete were evaluated using accelerated drying shrinkage, freeze–thaw cycling, sulfate wet–dry cycling, chloride penetration, optical microscopy, and normalised multi-criteria analysis. Based on the experimental results, the following conclusions can be drawn:

(1) Increasing SCM replacement generally increased drying shrinkage, whereas PVA fibres reduced shrinkage. The effects of SCMs and PVA fibres varied among the durability indicators. Increasing SCM replacement substantially reduced chloride penetration, while PVA fibres showed a clearer effect on compressive strength loss during freeze–thaw cycling. At 15%, 25%, and 35% SCM replacement, chloride penetration depth decreased by 26.7%, 33.3%, and 56.7%, respectively, relative to the control mixture.
(2) The effects of SCMs and PVA fibres differed among the durability indicators. SCM replacement was associated with lower chloride penetration, whereas PVA fibres showed a clearer effect on compressive strength retention during freeze–thaw cycling. At 25% SCM replacement, PVA fibres reduced freeze–thaw-induced compressive strength loss by 62.12–98.48% relative to the corresponding fibre-free mixture. This difference indicates that SCM replacement and PVA fibre incorporation affected the measured durability indicators differently.
(3) Optical microscopy revealed differences in visible pore-structure homogeneity among the specimens. A distinct internal stratified region was observed in one of the three C0.3 specimens but not in the other two specimens. This observation was therefore considered a local structural feature rather than a systematic characteristic of the mixture. The microscopy observations suggest that visible pore-structure uniformity may be relevant to FC performance, but they do not establish a direct causal relationship between the observed local stratification and the relatively high shrinkage of C0.3.
(4) The normalised multi-criteria evaluation showed that mixture ranking depended on the relative weighting of the five performance indicators. Under equal weighting, C0.3 achieved the highest overall performance score. Sensitivity analysis showed that changing the weighting scheme could alter the ranking, although C0.3 remained among the top three mixtures under all six weighting schemes considered. Within the investigated mixture range, C0.3 (25% SCM replacement and 0.3 vol.% PVA fibres) showed the most consistently high overall performance under the present evaluation framework, rather than representing a universally optimal mixture.
(5) The results should be interpreted within the adopted test conditions. Drying shrinkage was evaluated using an accelerated 60 °C drying procedure, while freeze–thaw and sulfate wet–dry resistance were assessed using 15 cycles. These tests provide comparative evidence of mixture performance but do not represent long-term durability under field conditions. Further research should evaluate these SCM–PVA fibre combinations using longer-term exposure, standardised durability protocols, and advanced microstructural characterisation to clarify the relationships between pore structure, moisture transport, cracking, and long-term durability.

## Figures and Tables

**Table 1:** Main oxide composition of OPC, FA, and GGBS.

Oxide Components	OPC	FA	GGBS
Al_2_O_3_ (%)	4.90	36.8	15.04
SiO_2_ (%)	22.16	45.1	33.06
CaO (%)	63.70	4.5	39.29
SO_3_ (%)	2.62	1.2	1.9
Fe_2_O_3_ (%)	3.48	-	-
MgO (%)	2.90	-	9.96
unreported oxide components (%)	0.24	12.4	0.75

Note: The test report provides the main oxide components listed above. Unreported oxide components are calculated by subtracting the reported main oxide content from 100%. Loss on ignition, chloride, alkali content, total iron content, etc., are not included in the total oxide count to avoid double counting.

**Table 2:** Mix proportions of FC.

Mix ID	OPC kg·m^−3^	FA kg·m^−3^	GGBS kg·m^−3^	Fibre kg·m^−3^	Water kg·m^−3^	PCE kg·m^−3^	CF kg·m^−3^	HPMC kg·m^−3^	Foam kg·m^−3^
A0	833.30	0.00	0.00	0.00	333.32	1.67	6.18	0.83	6.18
B0	708.30	62.50	62.50	0.00	333.32	1.67	6.18	0.83	6.18
C0	624.90	104.20	104.20	0.00	333.32	1.67	6.18	0.83	6.18
D0	541.70	145.80	145.80	0.00	333.32	1.67	6.18	0.83	6.18
A0.1	833.30	0.00	0.00	1.24	333.32	1.67	6.18	0.83	6.18
B0.1	708.30	62.50	62.50	1.24	333.32	1.67	6.18	0.83	6.18
C0.1	624.90	104.20	104.20	1.24	333.32	1.67	6.18	0.83	6.18
D0.1	541.70	145.80	145.80	1.24	333.32	1.67	6.18	0.83	6.18
A0.2	833.30	0.00	0.00	2.48	333.32	1.67	6.18	0.83	6.18
B0.2	708.30	62.50	62.50	2.48	333.32	1.67	6.18	0.83	6.18
C0.2	624.90	104.20	104.20	2.48	333.32	1.67	6.18	0.83	6.18
D0.2	541.70	145.80	145.80	2.48	333.32	1.67	6.18	0.83	6.18
A0.3	833.30	0.00	0.00	3.72	333.32	1.67	6.18	0.83	6.18
B0.3	708.30	62.50	62.50	3.72	333.32	1.67	6.18	0.83	6.18
C0.3	624.90	104.20	104.20	3.72	333.32	1.67	6.18	0.83	6.18
D0.3	541.70	145.80	145.80	3.72	333.32	1.67	6.18	0.83	6.18

Note: A–D denote SCMs replacement levels of 0%, 15%, 25%, and 35%, respectively, while the numerical suffixes 0, 0.1, 0.2, and 0.3 denote PVA fibre volume fractions of 0%, 0.1%, 0.2%, and 0.3%, respectively. For instance, B0.1 represents a mixture with 15% SCMs replacement and 0.1% PVA fibres by volume.

**Table 3:** Overall performance scores of the 16 mixtures under different weighting schemes.

Weighting Scenario	Equal Weighting 0.2	Drying Shrinkage 0.40	FT Strength Loss 0.40	FT Mass Loss 0.40	Sulfate Resistance 0.40	Chloride Penetration 0.40
A0	0.346	0.364	0.262	0.299	0.437	0.368
A0.1	0.579	0.577	0.437	0.685	0.662	0.536
A0.2	0.617	0.713	0.471	0.669	0.684	0.548
A0.3	0.490	0.485	0.376	0.567	0.594	0.426
B0	0.504	0.495	0.384	0.532	0.583	0.526
B0.1	0.478	0.476	0.367	0.483	0.574	0.488
B0.2	0.469	0.471	0.361	0.464	0.544	0.504
B0.3	0.499	0.517	0.396	0.509	0.582	0.493
C0	0.491	0.504	0.372	0.469	0.580	0.531
C0.1	0.485	0.513	0.372	0.480	0.572	0.485
C0.2	0.540	0.562	0.424	0.515	0.655	0.542
C0.3	0.683	0.675	0.762	0.606	0.745	0.626
D0	0.569	0.547	0.430	0.555	0.636	0.677
D0.1	0.457	0.466	0.346	0.448	0.556	0.467
D0.2	0.530	0.530	0.402	0.527	0.648	0.545
D0.3	0.479	0.497	0.363	0.467	0.599	0.469

## Data Availability

The original contributions presented in this study are included in the article. Further inquiries can be directed to the corresponding author.

## Abbreviations

The following abbreviations are used in this manuscript:

FC	Foamed Concrete
FA	Fly Ash
GGBS	Ground Granulated Blast-furnace Slag
PVA	Polyvinyl Alcohol
CF	Calcium Formate
HPMC	Hydroxypropyl Methylcellulose
PCE	Polycarboxylate Ether
OPC	Ordinary Portland Cement
PFA	Protein-based Foaming Agent
